# A THz Waveguide Bandpass Filter Design Using an Artificial Neural Network

**DOI:** 10.3390/mi13060841

**Published:** 2022-05-27

**Authors:** Chu-Hsuan Lin, Yu-Hsiang Cheng

**Affiliations:** Graduate Institute of Communication Engineering, National Taiwan University, Taipei City 10617, Taiwan; r08942015@ntu.edu.tw

**Keywords:** terahertz, waveguide bandpass filter, artificial neural network

## Abstract

This paper presents a 300 GHz waveguide bandpass filter based on asymmetric inductive irises. The coupling matrix synthesis technique is used to design a 6-pole Chebyshev filter. In addition, an artificial neural network is applied to provide the filter geometries using the desired frequency response. The optimized filter is fabricated by the computer numeric controlled milling process. The measurement results show that the insertion loss is less than 3 dB and the return loss is better than 17 dB in the range 276–310 GHz.

## 1. Introduction

As 5th generation wireless systems have become commercially available, researchers are studying higher frequency communications to further increase the data rate and reliability. The next frequency band higher than the current popular millimeter-wave frequency band is the terahertz frequency band (THz), which is usually defined as the frequency range from 300 GHz to 3 THz. When the operating frequency is increased to the terahertz waveband, metal loss, and surface roughness will have a great influence on the insertion loss. The development of low-loss terahertz filters is essential for wireless communication systems.

Since the wavelength of terahertz waves is much shorter than that of microwaves, the finest structures in the filters often require micrometer precisions to have good performances. Thus, sophisticated structures are difficult to be fabricated in the terahertz band. Currently, terahertz bandpass filters are mostly based on waveguide structures or metamaterial structures [1]. Due to the advantages of low loss, high power capacity, and high reliability, rectangular waveguides are particularly suitable for designing terahertz bandpass filters. Various fabrication technologies for the waveguide filters have been reported such as deep reactive ion etching processes [2], photolithography processes [3,4], and computer numerical control (CNC) milling processes [5,6,7,8], etc.

In the typical microwave design procedure, the geometries are first obtained by analytical methods and further optimized by numerical modeling to achieve the target response. In recent years, artificial neural networks (ANN) have been widely applied to speed up or to optimize the design process. As a common approach, ANN may take the device geometries as inputs and gives the S parameters as outputs, mimicking the electromagnetic simulation process [9,10]. Once the neural networks are developed, the computation time becomes much faster than the electromagnetic simulator, therefore, it is particularly suitable for designing and optimizing microwave component designs. In addition, ANN can also be used to solve the inverse design problem; the inputs are the desired frequency response and the outputs are the geometries of the microwave component [11,12].

In this paper, we present the design procedure as well as the measurement results of an asymmetric inductive iris-coupled waveguide bandpass filter in the 300 GHz band, which is fabricated by the CNC milling technology. In Section 2, we apply the coupling matrix synthesis technique to calculate the filter dimensions. In Section 3, an artificial neural network with the vector fitting technique is developed as an alternative way to obtain the filter dimensions. We have shown that the mean squared error of the dimensions is close to the CNC fabrication error. In Section 4, the optimized filter is fabricated and measured to prove the feasibility of the design. Finally, the conclusion is presented in Section 5.

## 2. The Coupling Matrix Synthesis Technique

We chose the 6th-order Chebyshev all-pole response for the proposed filter in order to achieve an equal ripple response in the passband and high out-of-band rejection. We designed the filter specification to match the Channel 67 of the IEEE 802.15.3d standard [13]; the filter can pass 278.64–304.56 GHz with a return loss greater than 25 dB in the passband.

**Figure 1:**
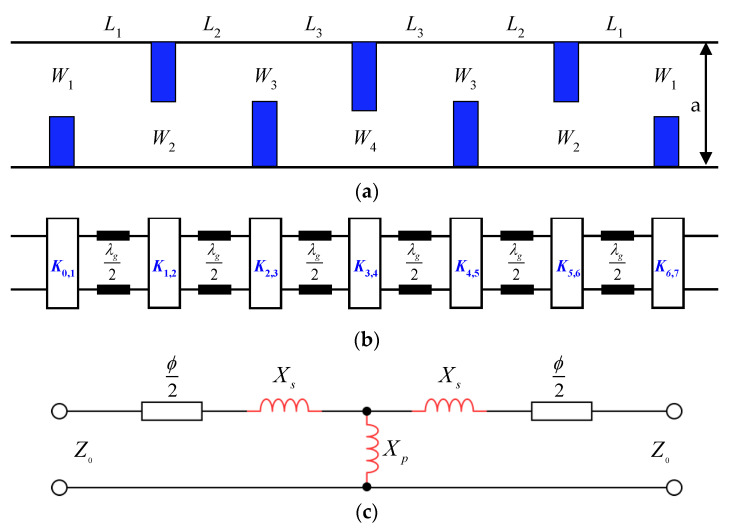
(**a**) Top view of the proposed 6th order waveguide filter with asymmetric irises; (**b**) Equivalent circuit of the filter in terms of impedance inverters; (**c**) The equivalent circuit of a K inverter.

The filter is based on a WR-3.4 standard rectangular waveguide (a = 864 μm and b = 432 μm). The filter structure and its equivalent circuit are shown in Figure 1. It consists of six waveguide resonators operating in the TE_101_ mode. The coupling between adjacent resonators is realized by asymmetric inductive irises. As shown in Figure 1, each inductive iris corresponds to an impedance inverter which is equivalent to a T-network with series and shunt reactance *X*_s_ and *X*_p_, and transmission lines of electric length *ϕ***/**2 on both sides. Considering that *ϕ* is negative, the final length of each resonator is slightly shorter than the half of the guided wavelength at the center frequency of the filter.

We followed the well-known design procedure, as shown in [14] and other references:

(1) Look up the lumped element values of the 6th Chebyshev lowpass prototype filter (0.01376 dB ripple):

*g*_0_ = 1, *g*_1_ = 0.821, *g*_2_ = 1.377, *g*_3_ = 1.729, *g*_4_ = 1.545, *g*_5_ = 1.541, *g*_6_ = 0.733, and *g*_7_ = 1.119.

(2) Calculate the normalized inverter *K_i,i_*_+ 1_ using the Chebyshev coefficients *g_i_*

(1) $$\frac{K_{i,i+1}}{Z_{0}}=\sqrt{\frac{\pi \Delta }{2g_{i}g_{i+1}}},\;i=0,6.$$

(2) $$\frac{K_{i,i+1}}{Z_{0}}=\frac{\pi \Delta }{2\sqrt{g_{i}g_{i+1}}},\;i=1\sim 5.$$

where Δ is defined as

(3) $$\Delta =\frac{\lambda_{gL}-\lambda_{gH}}{\lambda_{g0}}=0.138.$$

*λ_gL_*, *λ_gH_*, and *λ_g_*_0_ are the wavelength in the waveguide at the cutoff frequencies *f_L_*, *f_H_*, and center frequency *f*_0_.

**Figure 2:**
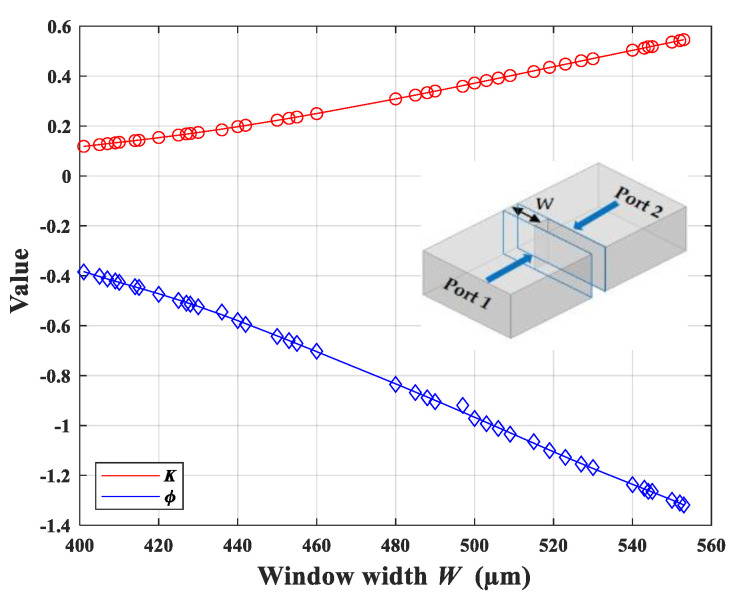
The relationship between the inverter value *K*, the electric length *ϕ*, and the window width *W* for an inductive iris in a WR-3.4 waveguide. The iris thickness is set to 100 μm. The inset shows the iris model and the de-embedded planes.

(3) We use the Ansys^®^ High-Frequency Structure Simulator (HFSS) to simulate each iris window. The ports are de-embedded to both sides of the iris. From the simulated S parameters, we can obtain *X_s_* and *X_p_*, and then determine the impedance inverter value *K*. Every iris thickness is set to 100 μm. We adjust the window width *W* so that the simulated *K* value at the central frequency becomes close to the designed value in step (2). The relation between the inverter value *K*, the electric length *ϕ*, and the window width *W* is shown in Figure 2, which also illustrates the importance of the fabrication accuracy.

(4) $$\frac{K}{Z_{0}}=\left|\tan\left(\frac{\phi }{2}+\arctan\frac{X_{s}}{Z_{0}}\right)\right|.$$

(5) $$\phi =-\arctan\left(2\frac{X_{p}}{Z_{0}}+\frac{X_{s}}{Z_{0}}\right)-\arctan\frac{X_{s}}{Z_{0}}.$$

(6) $$j\frac{X_{s}}{Z_{0}}=\frac{1-S_{12}+S_{11}}{1-S_{11}+S_{12}}.$$

(7) $$j\frac{X_{p}}{Z_{0}}=\frac{2S_{12}}{{\left(1-S_{11}\right)}^{2}-S_{12}^{2}}.$$

(4) Substitute Equation (5) into (8) to obtain the length of the resonator.

(8) $$L_{i}=\frac{\lambda_{g0}}{2\pi }\left[\pi +\frac{1}{2}\left(\phi_{i}+\phi_{i+1}\right)\right],\;i=0\sim 6.$$

Following the steps above, we obtain all the iris window widths *W* and resonator lengths *L*, which are summarized in Table 1.

## 3. Artificial Neural Network Modeling

Multilayer perceptron-based artificial neural networks have been used for microwave circuit design [15] as alternatives to the traditional design process. There are two different approaches to implement a neural network into the circuit design, the forward modeling network [16] and the inverse-design network [11,12]. We chose the inverse design approach that the artificial neural network outputs the filter dimensions with a given frequency response. We first generate the ground truth using HFSS simulations and then start the training process for the ANN that aims to reduce the error of the output filter dimensions. Once the training process is completed, we may input a certain filter response to the ANN and then obtain immediate results of filter dimensions without further electromagnetic simulations.

### 3.1. Vector Fitting Algorithm

In our case, the frequency sweep in the HFSS simulation is 260–340 GHz with a step of 0.1 GHz, therefore, there will be 801 frequency points. We do not use the S parameters at all frequency points as the inputs because a large number of inputs to the network would slow down the training process and make the process difficult to converge. In order to reduce the size of the network and speed up the training process, we apply the vector fitting [17] technique to fit the response of the S parameters by a model with poles and residues, and then feed those into the neural network for training.

Vector fitting algorithm is an order reduction technique. Consider a rational approximation function

(9) $$f(s)=\sum_{n=1}^{N}\frac{c_{n}}{s-a_{n}}+d+hs.$$

where *s* is the complex frequency, *N* is the fitting order which will control the accuracy of the approximation, *a_n_* is the n-th pole, *c_n_* is the n-th residue, *d* is the constant term, and *h* is the proportional term. In our case, *d* and *h* can be set to zero because our response does not have an asymptotic value. We thus simplify Equation (9) into

(10) $$f(s)=\sum_{n=1}^{N}\frac{c_{n}}{s-a_{n}}.$$

**Figure 3:**
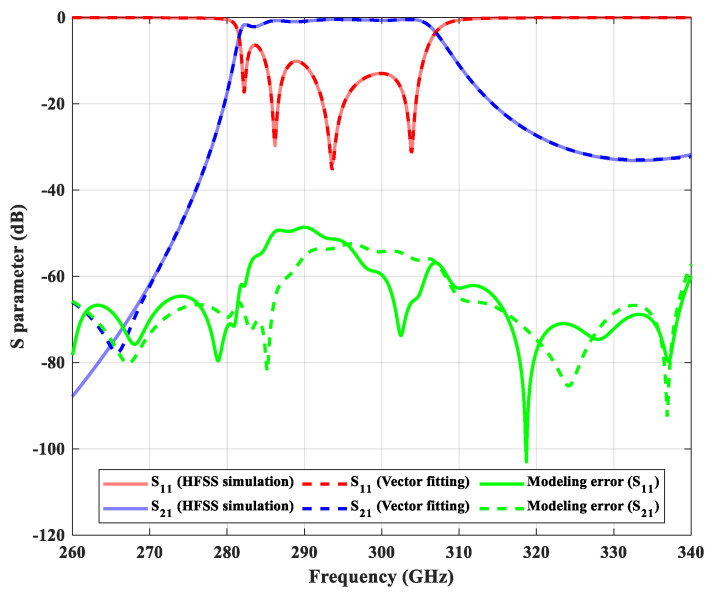
The comparison between the simulated S parameters of the filter and the corresponding vector fitting model.

The poles and residues can be obtained by solving the overdetermined linear equations iteratively [17]. We set the fitting order to 20 and thus reduce the 801 + 801 complex values down to 20 + 20 complex values for S_11_ and S_21_. As an example, we compare in Figure 3 the simulated S parameters and the corresponding vector fitting results for the filter designed in Section 2 (without optimization). Although the number of data points is greatly reduced, the frequency responses are very similar.

### 3.2. Artificial Neural Network Modeling

**Figure 4:**
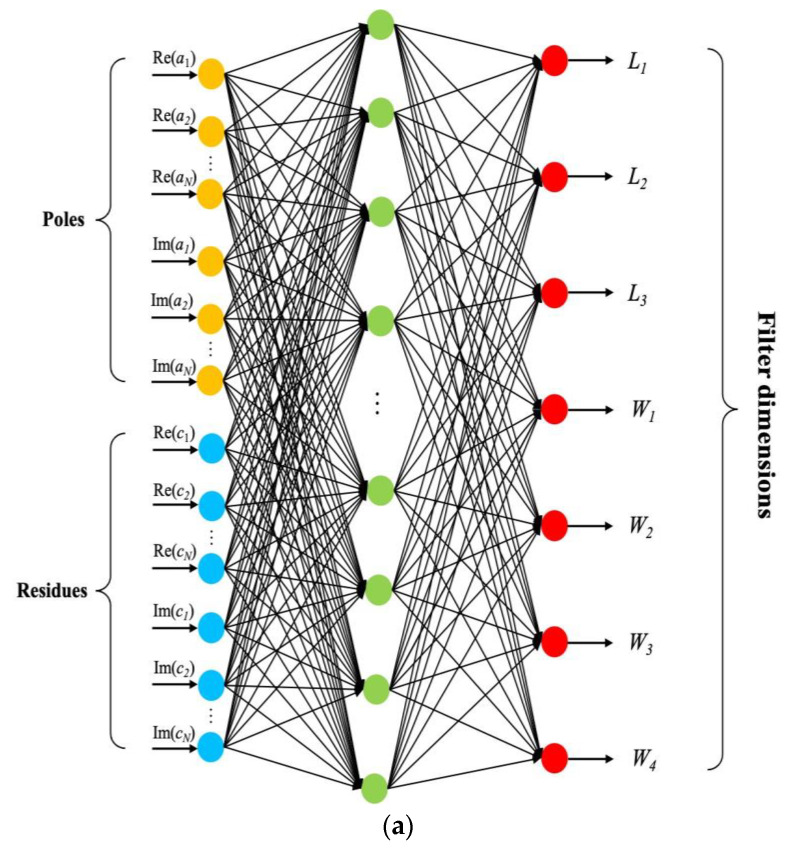

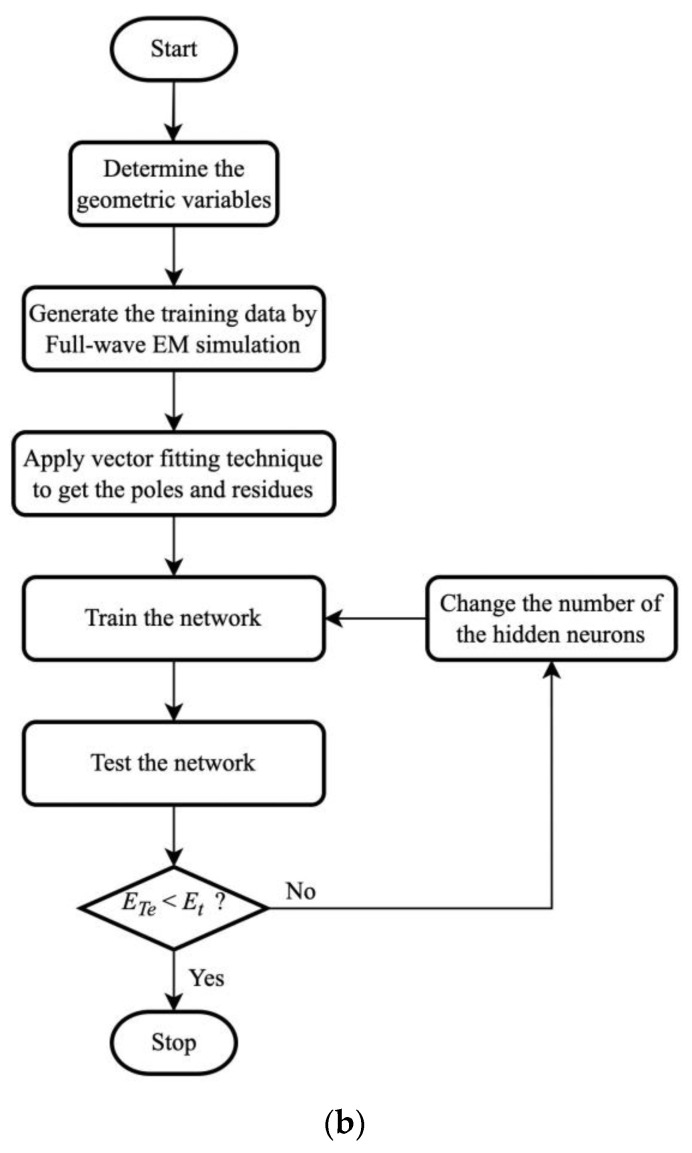
(**a**) The neural network structure of the proposed bandpass filter; (**b**) The flow chart of the overall process of the network model.

The inverse design neural network of the proposed bandpass filter is shown in Figure 4. Since the neural network does not support complex inputs, we divide the poles and residues into real and imaginary parts, and then feed them into the network. The number of neurons in the input layer is determined by the poles and residues found by vector fitting, and that in the output layer is determined by the filter dimensions. In our case, we have 80 neurons in the input layer and 7 neurons in the output layer. Numbers of neurons in the hidden layer are carefully adjusted to optimize the network structure. We choose the sigmoid to be the activation function and the scaled conjugate gradient algorithm for training [18]. The neurons in each layer are connected through a set of weights and biases. Training of the artificial neural network model is accomplished by adjusting these weights and biases to minimize the mean squared error between the HFSS simulation results and ANN modeling.

**Figure 5:**
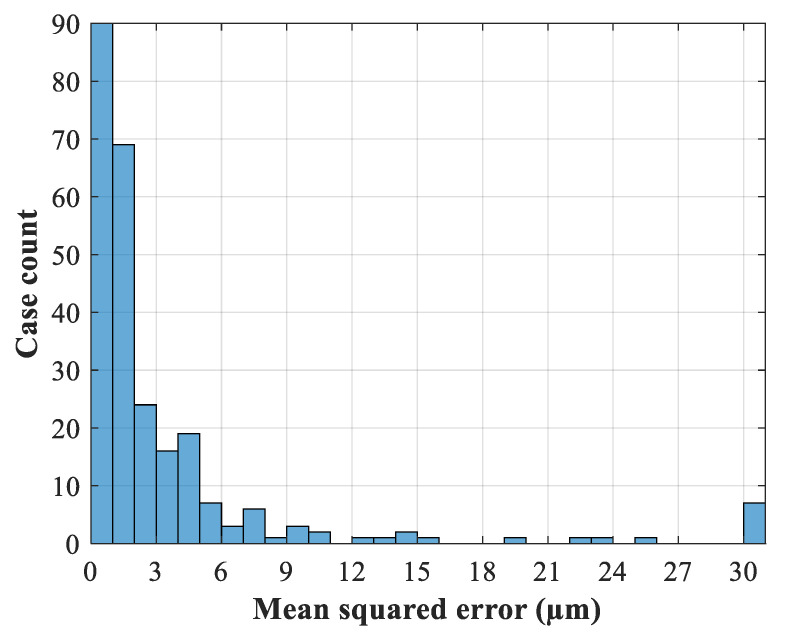
The histogram of the result of 256 test samples.

The data used for developing the artificial neural network is generated by HFSS simulations, and we apply the design of experiments [19] approach to generate the proper data. The number of samples and the ranges of the training data and test data are defined in Table 2. The flowchart in Figure 4 illustrates the overall process of the network model. The testing error *E_Te_* is defined as the mean squared error of all geometrical variables between the ANN modeling output and the testing data. If the testing error is lower than the error threshold *E_t_*, the process terminates and the network model is ready to use. The final number of neurons in the hidden layer after the training process is 40. Figure 5 shows the histogram of 256 test samples. The horizontal axis represents the mean squared error of the dimension parameters. As can be seen from the figure, there are 216 cases with an error less than 5 μm, close to the fabrication error of the CNC process.

Finally, the trained model is used to obtain the filter dimensions with the target response. The poles and residues of the target Chebyshev response, which is |S_11_| > 25 dB at the frequency range of 278.64–304.56 GHz, can be calculated without electromagnetic simulations. The predicted dimensions of the filter using the ANN are also summarized in Table 1. This ANN can be applied to design other filters with different poles and residues in the same frequency range. One can expand the range of the training dataset to increase the applicable frequency range of the ANN.

Before closing this section, we would like to compare the execution time of the coupling matrix synthesis method and the artificial neural network method. The execution time is estimated based on a computer with Intel I7-9700 CPU, 56 GB RAM, and NVIDIA GeForce RTX 2060 GPU. For coupling matrix synthesis, the main effort is to find out the relation between the window widths and the inverter values via HFSS simulation. The estimated execution time for coupling matrix synthesis is about 1 h. For the artificial neural network method, each training dataset takes about 5 min to generate using HFSS. The training and testing process can be completed within 30 min. Therefore, the estimated execution time for artificial neural network is about 64.5 h.

## 4. Fabrication and Measurement Results Analysis

### 4.1. Filter Structure

**Figure 6:**
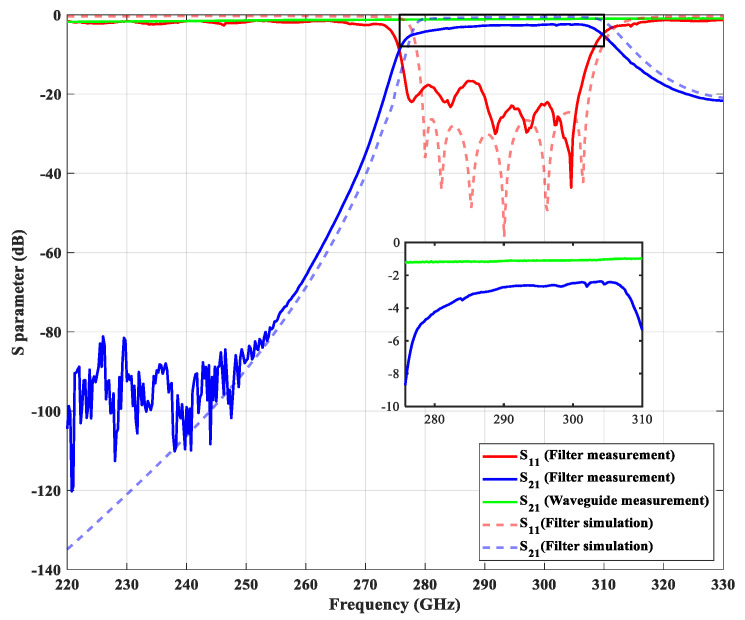
The simulation and measurement results of the proposed waveguide bandpass filter and the measured response of a straight waveguide for comparison.

**Figure 7:**
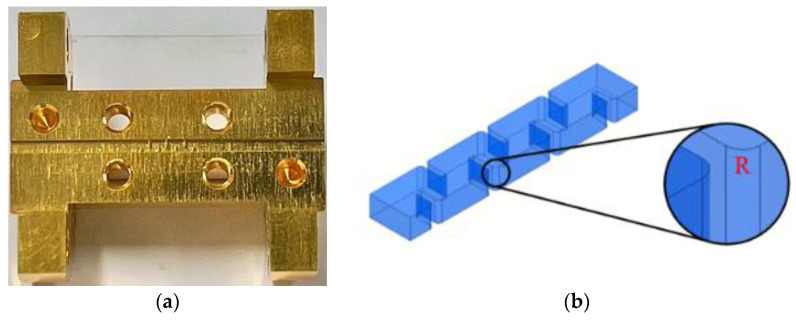

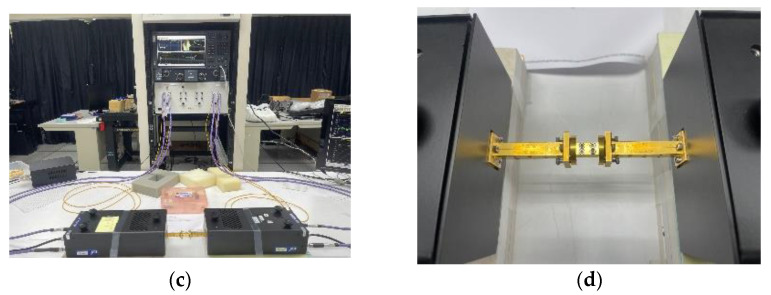
(**a**) Machining half blocks of the proposed THz 6-pole H-plane waveguide filter; (**b**) Geometric configuration. The inset shows the radius due to the CNC milling process; (**c**,**d**) The photos of the measurement setup.

The parameters obtained from the coupling matrix synthesis or the artificial neural network do not yet meet the filter requirement (|S_11_| > 25 dB in the passband). We set those parameters as the starting point and optimize the filter response using the HFSS software. The optimized dimensions of the filter are summarized in Table 1 and the simulated filter response is shown in Figure 6. A fillet of radius R = 75 μm is added to the edge of the iris to adapt to the requirement of CNC milling fabrication. In order to avoid misalignment, all geometric structures are machined in one of two metal blocks while the surface of the other metal block is kept flat, as shown in Figure 7.

### 4.2. Measurement Results

We measured the proposed filter at Taiwan Semiconductor Research Institute (TSRI) and the whole measurement environment is shown in Figure 7. The measurement was carried out by a vector network analyzer (Keysight N5242B PNA-X, Santa Rosa, CA, USA), a millimeter-wave controller (Keysight N5262A, Santa Rosa, CA, USA), and two millimeter-wave frequency extenders (Virginia Diodes Inc., Charlottesville, VA, USA). We used the calibration kit (Virginia Diodes Inc., Charlottesville, VA, USA) to perform the Through-Reflect-Line calibration. As shown in Figure 6, the measurement result indicates that the center frequency is 292.28 GHz and the fractional bandwidth is 11.64% from 276 GHz to 310 GHz. The in-band insertion loss is about 3 dB for a 22 mm long waveguide filter and the return loss is better than 17 dB. We also measured a 22 mm long straight waveguide without filter structures for comparison. The insertion loss of the straight waveguide is about 0.91 dB. The performance comparison between the proposed waveguide bandpass filter and other references are summarized in Table 3.

## 5. Conclusions

A THz waveguide bandpass filter with an asymmetric inductively coupled iris was designed, fabricated, and measured. We successfully applied both the traditional coupling matrix synthesis technique and the artificial neural network to design the filter. The execution time to generate training and testing datasets was the bottleneck to develop the artificial neural network. The measurement result indicated good in-band characteristics. This THz filter can be further integrated with other waveguide components such as horn antennas for wireless communication systems or other sensing applications.

## Figures and Tables

**Table 1:** Summary of dimensions of the waveguide filter, including the dimensions obtained by the coupling matrix synthesis technique, the dimensions from the artificial neural network, and the optimized dimensions using Ansys^®^ HFSS.

Coupling Matrix Synthesis						
*i*	$\frac{K_{i,i+1}}{Z_{0}}$	$\frac{X_{s}}{Z_{0}}$	$\frac{X_{p}}{Z_{0}}$	*ϕ_i_*_+1_	*W_i+_*_1_ (μm)	*L* (μm)
0	0.514	0.154	0.903	−1.252	543	*L*_1_ = 452 *L*_2_ = 534 *L*_3_ = 552
1	0.204	0.098	0.223	−0.595	442	
2	0.141	0.081	0.145	−0.444	414	
3	0.133	0.079	0.139	−0.421	409	
**Artificial neural network (unit: μm)**						
*W*_1_ = 568	*W*_2_ = 487	*W*_3_ = 417	*W*_4_ = 386	*L*_1_ = 445	*L*_2_ = 529	*L*_3_ = 531
**Optimized values (unit: μm)**						
*W*_1_ = 562	*W*_2_ = 471	*W*_3_ = 439	*W*_4_ = 431	*L*_1_ = 441	*L*_2_ =507	*L*_3_ = 531

**Table 2:** The range of the training and testing data.

Parameters	Training Data (512 Samples) (μm)	Testing Data (256 Samples) (μm)
*W*_1_	555–575	557–573
*W*_2_	460–480	462–478
*W*_3_	430–450	432–448
*W*_4_	420–440	422–438
*L*_1_	430–450	432–448
*L*_2_	500–520	502–518
*L*_3_	520–540	522–538

**Table 3:** Comparison of the state-of-the-art bandpass filters.

Ref.	*f*_0_ (GHz)	Order	Insertion Loss (dB)	Return Loss (dB)	Fractional Bandwidth	Techniques Employed
[20]	3.35	2	~2.4	>37	5.9%	SIW
[21]	1.68	3	~1.3	>22	4%	SIW
[22]	2.44	3	~0.1	>19.2	120%	Microstrip
[23]	4.83	4	~1.1	>10.5	131%	Microstrip
[3]	300	5	~1.6	>10	8%	SU-8 photoresist
[5]	298.6	3	~0.45	>16	5.36%	SU-8 photoresist
[5]	286	3	~0.41	>14	5.58%	CNC milling
[7]	255	5	~4	>15	12%	CNC milling
[8]	257.7	4	~0.7	>14	8.77%	CNC milling
[8]	256.3	4	~0.5	>15	9.83%	CNC milling
This work	292.28	6	~3	>17	11.64%	CNC milling

## Data Availability

The data presented in this study are available on request from the corresponding author.
